# PATZ1 in Non-Small Cell Lung Cancer: A New Biomarker That Negatively Correlates with PD-L1 Expression and Suppresses the Malignant Phenotype

**DOI:** 10.3390/cancers15072190

**Published:** 2023-04-06

**Authors:** Stefano Lucà, Renato Franco, Antonella Napolitano, Valeria Soria, Andrea Ronchi, Federica Zito Marino, Carminia Maria Della Corte, Floriana Morgillo, Alfonso Fiorelli, Antonio Luciano, Giuseppe Palma, Claudio Arra, Sabrina Battista, Laura Cerchia, Monica Fedele

**Affiliations:** 1 Pathology Unit, Department of Mental and Physical Health and Preventive Medicine, University of Campania “Luigi Vanvitelli”, 80138 Naples, Italy; 2 Institute for Experimental Endocrinology and Oncology (IEOS), National Research Council (CNR), 80145 Naples, Italy; 3 Department of Precision Medicine, Medical Oncology, University of Campania “Luigi Vanvitelli”, 80138 Naples, Italy; 4 Translational Medical and Surgical Science, Thoracic Surgery, University of Campania “Luigi Vanvitelli”, 80138 Naples, Italy; 5 S.S.D. Sperimentazione Animale, Istituto Nazionale Tumori—IRCCS—Fondazione G. Pascale, 80131 Naples, Italy

**Keywords:** PATZ1, lung cancer, PD-L1, tumor suppressor, LUSC, LUAD

## Abstract

**Simple Summary:**

Lung cancer is the leading cause of cancer death worldwide. Most lung cancers are classified as non-small cell lung cancer (NSCLC), which is diagnosed at an advanced stage when various treatments cannot be curative. Immunotherapy is a promising treatment for many cancers, including NSCLC, and the big challenge is the identification of new tumor biomarkers able to predict its success. In this framework, the aim of our study was to evaluate the expression of PATZ1, an emerging cancer-related protein suggested as a diagnostic marker in different cancers, in correlation with the expression of PD-L1, a major target of immunotherapy, in NSCLC. Our analysis, conducted in different NSCLC tumor samples and two NSCLC cell lines, indicated that PATZ1 and PD-L1 expressions are negatively associated and that PATZ1 overexpression downregulates PD-L1 expression. Furthermore, we show that deletion or halving of PATZ1 expression induces NSCLC in mice, whereas overexpression of PATZ1 in NSCLC cells reduces their ability to proliferate, migrate, and invade, compared to controls, suggesting that PATZ1 functions as a suppressor of NSCLC.

**Abstract:**

Non-small cell lung cancer (NSCLC), the leading cause of cancer death worldwide, is still an unmet medical problem due to the lack of both effective therapies against advanced stages and markers to allow a diagnosis of the disease at early stages before its progression. Immunotherapy targeting the PD-1/PD-L1 checkpoint is promising for many cancers, including NSCLC, but its success depends on the tumor expression of PD-L1. PATZ1 is an emerging cancer-related transcriptional regulator and diagnostic/prognostic biomarker in different malignant tumors, but its role in lung cancer is still obscure. Here we investigated expression and role of PATZ1 in NSCLC, in correlation with NSCLC subtypes and PD-L1 expression. A cohort of 104 NSCLCs, including lung squamous cell carcinomas (LUSCs) and adenocarcinomas (LUADs), was retrospectively analyzed by immunohistochemistry for the expression of PATZ1 and PD-L1. The results were correlated with each other and with the clinical characteristics, showing on the one hand a positive correlation between the high expression of PATZ1 and the LUSC subtype and, on the other hand, a negative correlation between PATZ1 and PD-L1, validated at the mRNA level in independent NSCLC datasets. Consistently, two NSCLC cell lines transfected with a PATZ1-overexpressing plasmid showed PD-L1 downregulation, suggesting a role for PATZ1 in the negative regulation of PD-L1. We also showed that PATZ1 overexpression inhibits NSCLC cell proliferation, migration, and invasion, and that Patz1-knockout mice develop LUAD. Overall, this suggests that PATZ1 may act as a tumor suppressor in NSCLC.

## 1. Introduction

Lung cancer is a heterogeneous group of diseases with respect to biology and clinical behavior and is one of the most common and aggressive cancers with the highest incidence and lethality. Indeed, the World Health Organization (WHO) has recently estimated a further increase worldwide in the coming years [1,2]. The most common lung cancer type is non-small cell lung cancer (NSCLC), representing about 85% of cases. It includes adenocarcinoma (LUAD), squamous cell carcinoma (LUSC), and large cell carcinoma, with LUAD and LUSC representing the largest NSCLC subgroups. The molecular pathogenesis of lung cancer involves the accumulation of genetic and epigenetic alterations, including the activation of proto-oncogenes and the inactivation of tumor suppressor genes, which differ among the lung cancer subgroups [3]. Overall, approximately 70% of lung cancer patients are diagnosed in advanced stages of the disease and are ineligible for tumor resection [3], prompting the identification of early diagnostic markers. Current treatment options for all NSCLCs include chemotherapy, radiotherapy, molecular targeted therapy, and immunotherapy, with subsets of patients treated according to the genetic alterations of their tumor and the status of programmed death ligand-1 (PD-L1), which predict for benefit from targeted therapies or immune checkpoint blockers, respectively [3,4]. However, LUSC and non-G12C KRAS mutant LUAD have no specific targetable therapeutic strategies to date [5].

Immune checkpoints are a strategic weapon of most cancer cells capable of inducing immunosuppression and consequently promoting tumor progression [6]. One of the major immune checkpoints is PD-L1, encoded by the CD274 gene, which can inhibit T cell priming against tumor antigens and induce tumor growth and progression in multiple tumors [7]. Aside from its tumor extrinsic role involving the tumor microenvironment, PD-L1 has a tumor intrinsic oncogenic role by inducing growth proliferation, migration, and invasive capability in several cancers including NSCLC [8].

PATZ1 (POZ/BTB and AT-hook-containing Zinc finger protein 1), also known as MAZR (MAZ Related factor), ZSG (Zinc finger Sarcoma Gene), or ZNF278 (Zinc finger protein 278), is an architectural transcription factor that regulates gene expression in a cell context-dependent manner by binding to gene promoter regions either directly, recognizing specific consensus sequences, or indirectly through its interaction with other proteins [9,10]. In particular, PATZ1 may be involved in the modulation of histone acetylation/deacetylation to maintain the transcriptional “off” state of different genes through interaction with corepressors and histone deacetylases, such as NCoR and SIRT1, respectively [11,12]. Consistently, Patz1^+/−^ mouse embryonic fibroblasts display epigenetic histone modifications characteristic of transcriptionally active chromatin [13]. PATZ1 expression is strongly related to cancer signatures and cellular proliferation, as recently assessed by PATZ1 ChIP-seq and gene expression microarray analyses [14]. Indeed, it has been shown to act as an oncogene in colon cancer and glioblastoma cells [15,16,17,18] and a tumor suppressor in glioblastoma [19], thyroid [20,21,22], ovarian [23], and liver cancer cells [14]. Additionally, PATZ1 has been proposed as a diagnostic/prognostic biomarker in different neoplasia, including testicular germ cell tumors [24], renal cell carcinoma [25,26,27], CNS neoplasms [28,29,30], round cell sarcomas [31], large cell B lymphomas [32], thyroid cancer [20], and ovarian cancer [23], where lower levels and cytoplasmic localization of the PATZ1 protein are often predictive of a more aggressive phenotype and, consequently, the worst prognosis [20,23,28,32]. The expression and role of PATZ1 in NSCLC still remain undetermined.

In the present study, we detected the expression of PATZ1 in NSCLC tissues, including LUSC and LUAD, by immunohistochemistry and bioinformatic analysis. Then, we evaluated the correlation of PATZ1 expression and localization with clinicopathological features, patient survival, and PD-L1 expression, showing a positive association with the LUSC subtype, where PATZ1 behaves as a favorable prognostic marker, and a negative association with PD-L1 expression, which suggests that PATZ1 may be involved in its negative regulation. On the other hand, nuclear expression of PATZ1 is negatively associated with the LUAD subtype, and Patz1-knockout mice develop lung adenocarcinomas. Consistently, overexpression of PATZ1 in LUAD cells inhibits cell proliferation, migration, and invasion, suggesting a tumor suppressor role of this gene in NSCLC, likely by counteracting the epithelial–mesenchymal transition.

## 2. Materials and Methods

### 2.1. Patient Cohorts and Public Datasets

A cohort of 104 histologically confirmed NSCLC specimens, including 43 LUSCs and 61 LUADs, were collected from the Department of Pathology of the Università degli Studi della Campania “L. Vanvitelli”, Naples, Italy, and evaluated retrospectively in this study. All cases were reviewed according to the most up-to-date WHO classification criteria, using standard tissue sections stained with hematoxylin/eosin and analyzed by immunohistochemistry.

Other cohorts were collected from publicly available datasets from the Gene Expression Omnibus (GEO) database and the Cancer Genome Atlas Program (TCGA). They include the GSE31552 dataset, from the Albert Einstein College of Medicine, NY, USA, from which we selected 50 NSCLC (23 LUSC and 27 LUAD) and 50 paired normal perilesional alveolar epithelial tissues for the analysis of PATZ1 expression in tumor vs. non-tumor tissues, and the whole dataset of 131 paired and unpaired tumor and non-tumor tissues for the analysis of the correlation between PATZ1 and SOX2 expression levels; the GSE19804 dataset, consisting of 60 pairs of tumor and adjacent normal NSCLC tissue specimens from nonsmoking women in Taiwan, and the GSE33532 dataset, including 4 different sites of individual primary tumors and matched distant normal lung tissue from 20 patients with early-stage NSCLC in Heidelberg, Germany, for the analysis of the correlation between expressions of PATZ1 and either CD274, CDH1, or VIM; the GSE10072 dataset, including 58 LUAD samples and 49 non-tumor adjacent tissues collected from several hospitals in Italy, for correlation analyses between expressions of PATZ1 and either CDH1, VIM, or SOX2. From the TCGA, we used a dataset of 515 LUAD samples for overall survival and correlation analysis between PATZ1 and CD274; a dataset of 81 LUSC samples for the analysis of the correlation between PATZ1 and SOX2 expression levels; and metadata from 994 patients, including 494 with LUSC and 500 with LUAD, for survival analysis. Finally, an integrated database from the Cancer Biomedical Informatics Grid (caBIG), GEO, and TCGA repositories [32] was used for overall survival (OS) analysis in 524 LUSC patients and progression-free survival (PFS) after immunotherapy in 463 PAN-cancer patients, through the Kaplan–Meier platform [33].

### 2.2. Tissue-Microarray and Immunohistochemical Study

The tumor area of the preliminarily selected NSCLC samples was cored for the development of the Tissue Micro Array (TMA). TMA building and immunohistochemical staining was performed as previously described [34]. Primary antibodies were directed to PATZ1 (custom polyclonal antibody R1P1, Primm, Milano, Italy) and PD-L1 (monoclonal antibody SP263, Ventana Medical Systems, Tucson, AZ, USA). The primary antibody against PATZ1 is a previously described rabbit polyclonal antibody [20,28,34] raised against a peptide in the N-terminal region of the human PATZ1 protein (aa 1-276). Immunohistochemistry in mouse tissues was performed using the avidin–biotin–peroxidase LSAB+ kit (Dako, Glostrup, Denmark) on 5 μm thick sections as previously described [24]. Briefly, endogen peroxidases were quenched by incubation in 0.1% sodium azide with 0.3% hydrogen peroxide for 30 min at room temperature. Non-specific binding was blocked by incubation with nonimmune serum. The antibodies used were E-cadherin (24E10) Rabbit mAb #3195 (Cell signaling, Danvers, MA, USA) diluted 1:400, N-cadherin (D4R1H) XP Rabbit mAb #13116 (Cell signaling) diluted 1:100, vimentin (D21H3) XP Rabbit mAb #5741 (Cell signaling) diluted 1:200, and PD-L1 (E1L3N) XP Rabbit mAb #13684 (Cell signaling) diluted 1:200.

### 2.3. Cell Culture, Transfections, and PATZ1 Subcellular Localization

A549 and H1299 human LUAD cells were obtained from the American Type Culture Collection (Rockville, MD, USA) and grown in Dulbecco’s Modified Eagle Medium (DMEM, with 4500 mg glucose/L, 110 mg sodium pyruvate/L, and L-glutamine, Sigma, St. Louis, MO, USA) supplemented with 10% fetal bovine serum (FBS) (Sigma) and 1% penicillin–streptomycin. All cells were cultivated at 37 °C and 5% CO_2_ and were split every 2–3 days.

Transfections were performed using jetPRIME^®^ (Polyplus transfections, Illkirch, France), with pHa-PATZ1 plasmid or the empty vector pCEFL-HA as a negative control [10], following the manufacturer’s instructions. At 5 h after transfection, cells were harvested, washed twice in PBS, and plated for the different assays.

For PATZ1 subcellular localization, the PATZ1-EGFP-C2 plasmid, carrying the PATZ1 cDNA fused with the enhanced green fluorescent protein [20], was transfected as above. Green fluorescence (485 nm excitation, 510 nm emission wavelengths) was measured on a Nikon eclipse Ti2 microscopy using the NIS-elements AR software (Nikon Instruments Inc., Melville, NY, USA). To further assess the nuclear localization of the PATZ1-EGFP protein, cells that were previously seeded on a coverslip for 24 h were fixed in 4% paraformaldehyde (PFA) for 20 min at room temperature and stained for 5 min at RT with 1.5 μM 4′,6-Diamidino-2-phenylindole (DAPI, D9542, Sigma). After three washes with Dulbecco’s phosphate-buffered saline (DPBS), coverslips were mounted with glycerol/DPBS. Samples were visualized by green fluorescence (then converted in red on the images), for the PATZ1-EGFP protein, and blue fluorescence (359 nm excitation, 457 nm wavelengths), for DAPI-stained nuclei, using Zeiss LSM 700 META confocal microscopy equipped with a Plan-Apochromat 63×/1.4 Oil DIC objective.

### 2.4. Cell Viability, Tripan Blue Exclusion, and Colony Formation Assays

Cells plated in 96-well plates (5000/well) were analyzed for cell viability at 24 h after transfection (Time 0) and in the following 24 h and 48 h by using CellTiter96 AQueous Non-radioactive Assay (Promega, Milano, Italy), following the manufacturer’s instructions. To assess the presence of dead cells, nonadherent and adherent cells were collected, and aliquots were mixed with an equal volume of 0.4% trypan blue (Life Technologies, Monza, Italy). All cells that incorporated the blue color were counted as dead cells. For the colony formation assay, cells were seeded out in appropriate dilutions to form colonies in 2 weeks. During this period, the transfected cells were selected with a standard medium containing 800 μg/mL G418 (D.B.A. Segrate, MI, Italy). G418-resistant colonies were fixed with methanol (25% *v*/*v*) and stained with crystal violet (0.1% *w*/*v*). The crystal violet was eluted with 10% SDS, and the absorbance was measured at 594 nm. Data are representative of at least three independent culture dishes per time point.

### 2.5. Cell Migration and Invasion Assays

The migratory capacity of cells was analyzed using Transwell^®^ inserts with 8.0 μm pore size (AlfaMed, Giugliano, NA, Italy). To the upper compartments, 50,000 cells in 0.1 mL of serum-free medium were added, while the lower compartments were filled with 0.6 mL of DMEM containing 10% FBS as a source of chemoattractants. The inserts were incubated for 48 h at 37 °C and 5% CO_2_. After incubation, cells were fixed in methanol (25% *v*/*v*) and stained with 600 µL crystal violet (0.1% *w*/*v*) for 30 min at 4 °C. Cells on the top surface of the filters were wiped off with cotton swabs. For A549, cells that had migrated into the lower compartment and attached to the lower surface of the filter were counted under the microscope. The migration rate was expressed as number of migrated cells per field of view. Alternatively, for H1299 cells, crystal violet was eluted in 10% SDS, and the absorbance was measured at 594 nm. Data are representative of at least three independent culture dishes per time point.

For the invasion assay, Matrigel was thawed and liquefied on ice at 4 °C for about 5 h. The upper chambers of 24-well transwell inserts were coated with 50 μL of a mixture of serum-free medium and Matrigel (6:1; Euroclone, Pero, MI, Italy) and allowed to solidify at 37 °C for minimum 45 min. Cells were re-suspended in 100 µL of serum-free cell culture media, plated (100,000/well) on top of the filter membrane, and incubated for 48–72 h at 37 °C in 5% CO_2_. The lower chambers were filled with 500 μL DMEM containing 10% FBS for H1299 and 20% FBS for A549. The quantification of migrated cells through the extracellular matrix was performed counting the number of cells in 5 randomly selected fields after fixing and staining with methanol 25%/crystal violet 0.1% for 15 min, washing with PBS and drying off.

### 2.6. RNA Extraction, Reverse Transcription, and RTqPCR

Total RNA was extracted from cells using TRI-reagent solution (Sigma) following the manufacturer’s protocol. Reverse transcription was performed according to standard procedures using a SensiFAST™ cDNA Synthesis Kit (Aurogene, Rome, Italy). An Applied Biosystems 7900 Real-Time PCR Detection System (ABI 7900HT—Applied Biosystems, Waltham, MA, USA) was used to amplify each cDNA with iTaq Universal SYBR Green Supermix (Bio-Rad, Hercules, CA, USA). The PCR conditions were as follows: 95 °C for 10 min followed by 40 cycles at 95 °C for 15 s and 60 °C for 1 min. Subsequently, the dissociation curve was calculated to verify the amplification specificity. Each amplification reaction was repeated in duplicate. Primer pairs used were as follows: PATZ1 all variants: 5′-TACATCTGCCAGAGCTGTGG-3′/5′-TGCA CCTGCTTGATATGTCC-3′; ꞵ-ACTIN: 5′-CAAGAGATGGCCACGGCTGCT-3′/5′-TCCTTCTGCATCCTGTCGGCA -3′; CDH1: 5′-GCCTCCTGAAAAGAGAGTGGAAG-3′/5′-TGGCAGTGTCTCTCCAAATCCG-3′; VIM: 5′-GACCAGCTAACCAACGACAAA-3′/5′-GAAGCATCTCCTCCTGCAAT-3′.

The 2^−ΔΔCT^ method was used to calculate the gene expression levels [35]. Beta-ACTIN was used as the internal normalizer for gene expression.

### 2.7. Protein Extraction, Western Blotting, and Antibodies

Harvested cells were lysed in high-salt extraction buffer (10 mM Hepes pH 7.8, 400 mM NaCl, 0.1 EGTA, 0.5 mM DTT, 5% glycerol, and 0.5 mM PMSF) supplemented with 25% proteinase inhibitor cocktail (Complete Mini—Roche, Basel, Switzerland). The concentration of proteins in cell lysates was quantified by the Bio-Rad Protein Assay Dye Reagent Concentrate (Bio-Rad), and 40 μg protein was loaded in each lane. Samples were electrophoresed with SDS–PAGE (10%) and blotted for 1 h onto PDVF membranes (Immobilon-P, Millipore, Milano, Italy). The membranes were activated with methanol (100%) and blocked with 5% non-fat milk for 1 h at room temperature (RT). Blots were incubated with the primary antibodies overnight at 4 °C, and then with the secondary antibodies for 1 h at RT, before being visualized by Clarity Western ECL Substrate (Bio-Rad). Five to seven washes in TTBS buffer were done after each antibody incubation. The antibodies used were as follows: anti-PATZ1 [20]; anti-PD-L1 (Cell Signaling, Danvers, MA, USA #13684, 1:1000); anti-vimentin (Cell Signaling, D21H3, 1:1000); anti-vinculin (Santa Cruz, Dallas, TX, USA, sc-73614, 1:1000); HRP-conjugated goat-anti-rabbit IgG (Santa Cruz, sc-2004, 1:2000); and HRP-conjugated goat-anti-mouse IgG (Santa Cruz, sc-2005, 1:2000).

### 2.8. Statistical Analysis and Kaplan–Meier Survival Curves

Pearson’s χ^2^ test was used to evaluate the correlations between the PATZ1 score and the clinical variables (age, gender, grade, subtype, metastases). All correlations within the publicly available datasets were assessed by one-way analysis of variance (ANOVA), through the R2: genomic Analysis and Visualization platform [36].

Kaplan–Meier survival curves were used to analyze OS/PFS, and statistical significance was assessed by the log-rank test. The stratification of High/Low PATZ1 in the analysis of public datasets was based on RNA expression resulting from the scan expression function of the above mentioned R2 platform, where the best expression cut off is established based on statistical testing (log-rank test).

All other statistical analyses were performed using GraphPad Prism 6 software (GraphPad Software, La Jolla, CA, USA). Each experiment was independently repeated three times, and the results are presented as the mean ± standard error (SE). A *t*-test was performed to determine the differences between two groups. A probability (*p*) value less than 0.05 was considered statistically significant.

### 2.9. Animals

Patz1-knockout mice and their pathological phenotype were previously described [9,37]. Briefly, the *Patz1* gene targeting vector was derived from a λΦXII phage library of a 129SvJ mouse strain (Stratagene, La Jolla, CA, USA). It was designed to delete a 2317-bp PstI-XhoI fragment including the start codon, the coding region for the POZ domain, the AT-hook, and the first four zinc fingers. The targeting construct was electroporated into 129SvJ-derived embryonic stem cells (ES). Two correctly targeted ES clones were injected into C57Bl/6J blastocysts. Both gave rise to germ line chimeras that were backcrossed to C57Bl/6J females to obtain heterozygous Patz1-knockout (ko) offspring. All Patz1-ko mice and their wild-type controls were maintained, in a mixed 129SvJ/C57Bl/6J genetic background, under standardized non-barrier conditions in the Laboratory Animal Facility of the Istituto dei Tumori di Napoli. All studies were conducted in accordance with Italian regulations for experimentations on animals. For histologic examination, dissected tissues were fixed by immersion in 10% formalin and embedded in paraffin. Mounted sections (5 μm thick) were stained with hematoxylin and eosin using routine procedures.

## 3. Results

### 3.1. Tissue Micro-Array Design and Clinicopathological Data

One hundred and four NSCLC tissue samples were used for TMA building, using three tissue cores taken from two to three discrete but representative regions of each individual case. The main clinicopathological data included in the TMA are set out in Table 1. The series included 29 (27.9%) women and 75 (72.1%) men, with a median age of 65.6 ± 2.77 years. In our histological samples, there were 43 (41.3%) LUSCs and 61 (58.7%) LUADs. Most women (72.4%) developed LUAD rather than LUSC, while men developed LUAD or LUSC at almost the same frequency. Indeed, 81.4% of LUSCs were diagnosed in men, consistent with most recent studies showing men are more likely to get LUSC [38]. Regarding tumor cell differentiation, both LUSCs and LUADs in the TMA included well-differentiated (G1), moderately differentiated (G2), and poorly differentiated (G3) tumors in the percentages indicated in Table 1.

### 3.2. PATZ1 Is Differently Expressed and Mislocalized in NSCLC

**Figure 1:**
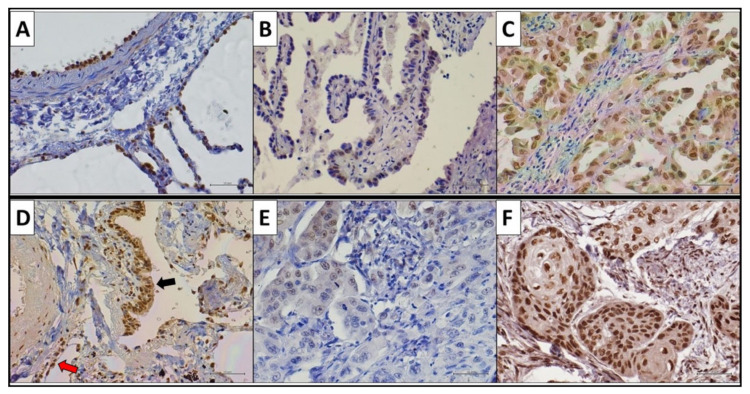
Immunohistochemical PATZ1 expression in NSCLC and peritumoral tissue. (**A**–**C**) Representative staining of PATZ1 in LUAD samples and peritumoral tissue. (**D**–**F**) Representative staining of PATZ1 in LUSC samples and peritumoral tissue. (**A**) PATZ1 nuclear expression in normal pneumocytes; (**B**) PATZ1 nuclear expression in <20% of LUAD cells (low-expressor LUAD); (**C**) PATZ1 nuclear expression in ≥70% of LUAD cells (high-expressor LUAD); (**D**) PATZ1 nuclear expression in normal pneumocytes (red arrow) and nuclear and cytoplasmic expression in bronchiolar cells (black arrow) with stronger nuclear staining; (**E**) PATZ1 nuclear expression in <20% of LUSC cells (low-expressor LUSC); (**F**) PATZ1 nuclear expression in ≥70% of LUSC cells (high-expressor LUSC). Scale bar: 50 μm.

We analyzed PATZ1 expression by immunohistochemistry in the TMA described above. In the normal peritumoral tissue (bronchiolar and alveolar), PATZ1 was expressed in a low percentage of cells (<20%) and was exclusively nuclear in the alveolar epithelium, while both nuclear and cytoplasmic in most of the bronchiolar tissues analyzed. Conversely, in LUAD and LUSC, PATZ1 expression was more heterogeneous, being nuclear, cytoplasmic, both cytoplasmic and nuclear, or absent (Figure 1 and Table 2). In particular, specimens with nuclear or nuclear/cytoplasmic expression of PATZ1 represented the great majority of LUSCs (72%), whereas PATZ1 protein was mostly delocalized in the cytoplasm in LUADs (76%) compared to LUSCs (53%) (Table 2).

Consistently, statistical analyses based on Pearson’s correlation proved that PATZ1 nuclear expression correlates positively with the LUSC subtype and negatively with the LUAD subtype (*p* < 0.01) (Table 3).

The cytoplasmic delocalization of PATZ1, which is suggestive of a loss of function of the protein, is consistent with previous results in testicular germ cell tumors, thyroid carcinomas, and large B cell lymphomas [20,24,34].

### 3.3. PATZ1 High Expression Correlates with the LUSC Subtype and Is a Favorable Prognostic Marker

Based on the percentage of PATZ1 positive cells, we divided our cohort of tumor samples in three groups, namely Low (absent or <20%), Medium (>20%; <70%), and High (≥70%) PATZ1 (Table 4). For each group, the staining of PATZ1 was nuclear, nuclear/cytoplasmic, or cytoplasmic.

Considering the group with the High PATZ1 score, we found a significant positive association with the LUSC subtype and a negative association with the LUAD subtype (Table 5).

**Figure 2:**
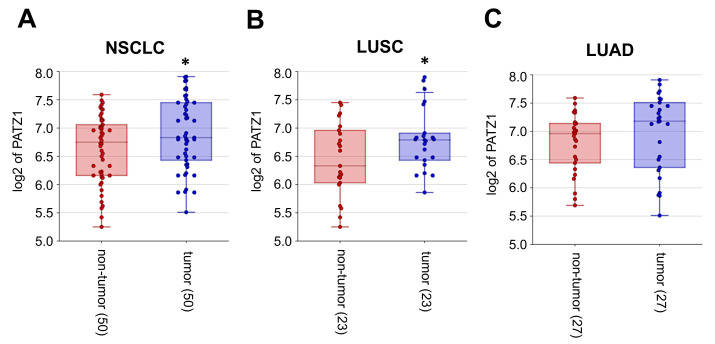
PATZ1 is overexpressed in tumor vs. non-tumor NSCLC samples. (**A**) A gene expression dataset of 50 pairs of tumor and non-tumor NSCLC (GSE3152), including 23 LUSC and 27 LUAD, was analyzed for the expression of PATZ1 via the R2: Genomic Analysis and Visualization platform [36]. (**B**) The same analysis was performed in the subset of LUSC and (**C**) LUAD samples. * *p* < 0.05.

Due to the limited number of adjacent normal tissue in our local cohort, it was not possible to statistically compare PATZ1 expression in tumor versus non-tumor tissue. However, by analyzing publicly available datasets through the R2: Genomic Analysis and Visualization platform [36], we confirmed, at the mRNA level in an independent cohort of 50 NSCLC (23 LUSCs; 27 LUAD) samples and paired non-tumor adjacent alveolar tissues [39], that PATZ1 is significantly overexpressed in tumor versus non-tumor tissues (Figure 2A). The significance is retained in the subgroup of LUSC (Figure 2B), but not in the subgroup of LUAD (Figure 2C).

**Figure 3:**
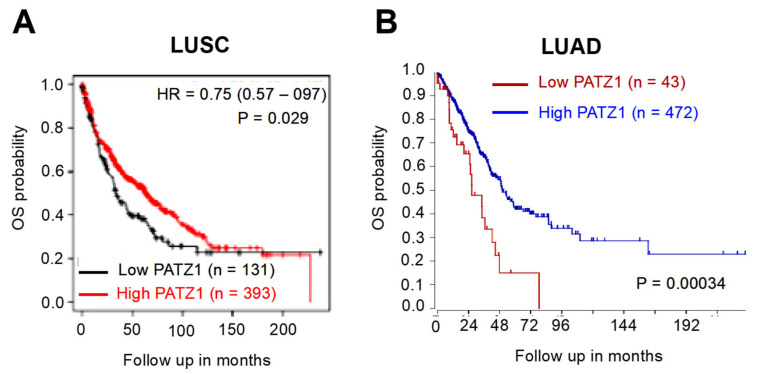
Low expression of PATZ1 gene is associated with a worse prognosis. (**A**) Overall survival (OS) metanalysis in LUSC based on PATZ1 expression using publicly available transcriptomic and clinical data via the Kaplan–Meier Plotter platform [33]. The expression cutoff was set to the lower quartile. (**B**) OS analysis in LUAD based on PATZ1 expression using a gene expression data subset from the TCGA via the R2: Genomic Analysis and Visualization platform [36]. The expression cutoff was set to 632 (lower sextile) according to the scan function. HR, Hazard ratio.

Interestingly, according to survival analyses performed with mRNA datasets of 524 LUSCs, publicly available via the Kaplan–Meier Plotter platform [32,33,40], and 515 TCGA LUADs, available through the R2: Genomic Analysis and Visualization platform, low levels of PATZ1 are associated with worse survival in both LUSC and LUAD (Figure 3). This suggests that although PATZ1 is overexpressed in lung cancer compared to normal lung tissue, higher expression of PATZ1 in the tumor is prognostic for a more favorable outcome. Indeed, LUSC patients with high PATZ1 expression had a median survival of 64.1 months compared with those with low PATZ1 expression, who had a median survival of 33.35 months (Figure 3A). Similarly, median survival in LUAD patients was 48 months and 24 months in the high and low PATZ1 cohorts, respectively (Figure 3B).

The favorable prognostic role of elevated PATZ1 expression in lung cancer was confirmed in an independent TCGA mRNA cohort of 994 lung cancer patients (494 LUSCs; 500 LUADs) via the Human Protein Atlas platform [41], in which the 5-year survival was 48% for the high PATZ1 group and 34% for the low PATZ1 group (*p* < 0.0001) (Figure S1).

### 3.4. PATZ1 Expression Negatively Correlates with PD-L1 Expression

PD-L1 (CD274) is highly expressed in various cancers, including NSCLC, where it plays an important role in tumor growth and progression [8]. In our local cohort of samples, PD-L1 (IHC, cut-off 1%) was expressed in 45 out of 85 NSCLCs (53%), and it was negatively associated with the High PATZ1 subgroup (Table 6).

**Figure 4:**
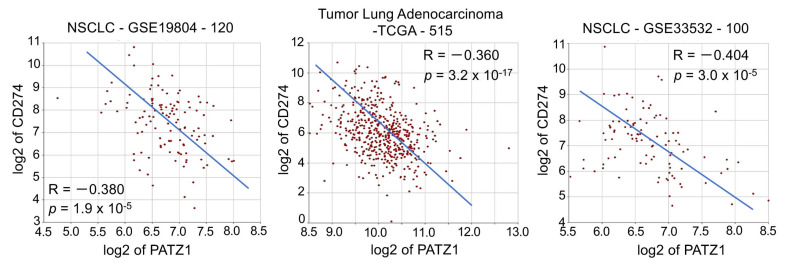
PATZ1 and CD274 expression are inversely correlated. XY-dotplots showing the correlation between PATZ1 (*X*-axis) and CD274 (*Y*-axis) gene expression in three publicly available NSCLC gene expression datasets analyzed via the R2: Genomic Analysis and Visualization platform [36].

The negative correlation between PATZ1 and PD-L1 was confirmed at the mRNA level in three independent NSCLC datasets publicly available via the R2: Genomic Analysis and Visualization platform [36] (Figure 4). Interestingly, albeit with lower R, a significant negative correlation was also observed between PATZ1 and the Programmed Cell Death 1 gene PD-1 (also named PDCD1) in LUADs, while it was not significant in NSCLCs, including both LUSCs and LUADs (Figure S2).

**Figure 5:**
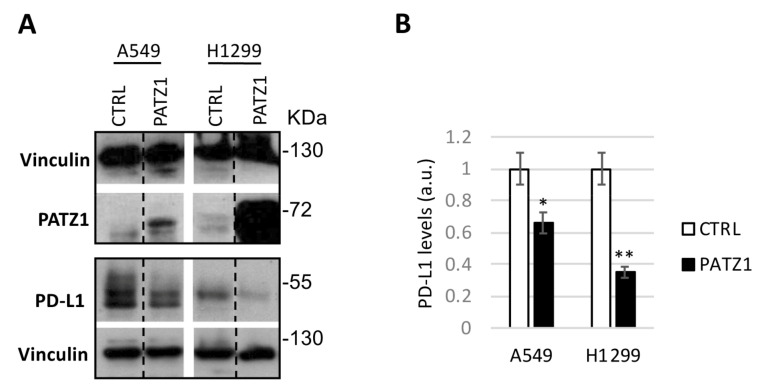
PATZ1 overexpression in lung adenocarcinoma cells reduces PD-L1 expression. (**A**) Western blots using anti-PATZ1 and anti-PD-L1 on total cell extracts of A549 and H1299 cells collected 24 h after transfection with PATZ1-overexpressing plasmid (PATZ1) or empty vector used as control (CTRL). Vinculin was used for normalization. Black dashed lines delineate the boundary between non-contiguous lanes of the same gel. (**B**) Relative PD-L1 levels were quantified by densitometric analysis using Image J (v1.46r), normalized relative to vinculin levels, and expressed relative to each control, arbitrarily set to 1. a.u., arbitrary units. * *p* < 0.05; ** *p* < 0.01. The uncropped blots are shown in Figure S3.

In order to investigate a possible role of PATZ1 overexpression in downregulating PD-L1 expression in NSCLC cells, we transiently transfected A549 and H1299 cells with a plasmid expressing the human PATZ1 gene and analyzed PATZ1 and PD-L1 protein levels 24 h later by Western blot (Figure 5). A relevant decrease of PD-L1 was observed in cells overexpressing PATZ1 compared to empty-transfected control, supporting an inhibitory role of PATZ1 on PD-L1 expression.

**Figure 6:**
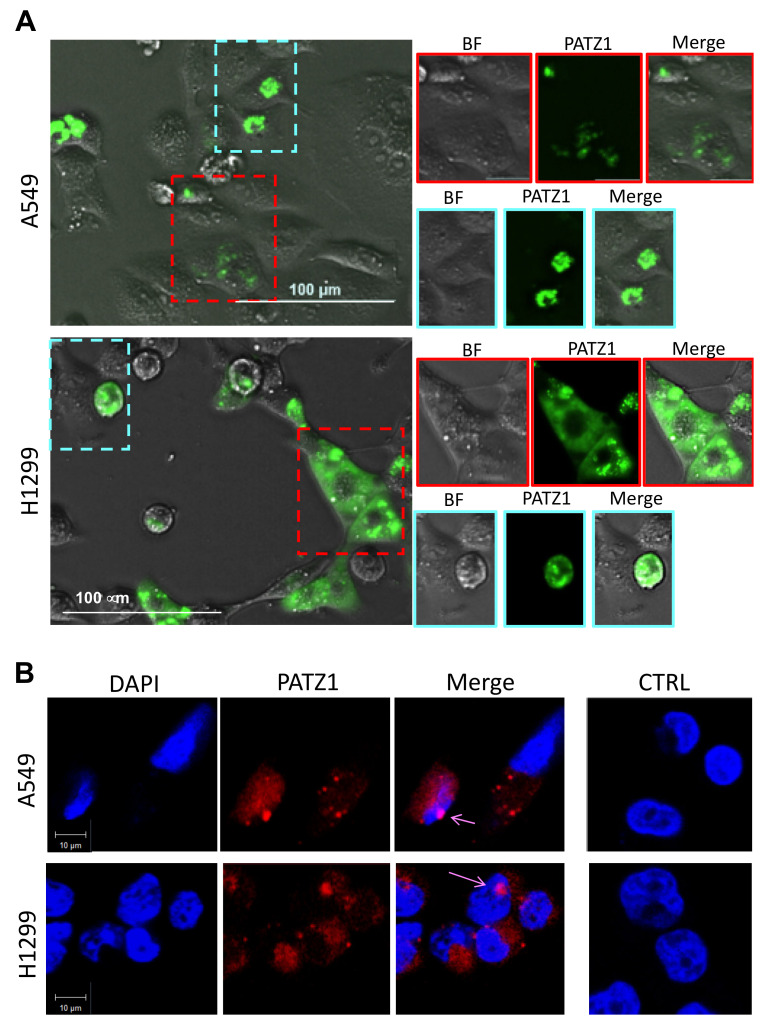
PATZ1 subcellular distribution in A549 and H1299 transfected cells. (**A**) Fluorescent microscopy in living cells showing the merging images of green fluorescent PATZ1 protein and bright field. Dotted frames enclose cells in which PATZ1 expression appears localized in the nucleus (cyan) or cytoplasm (red). Outsets on the right report each single bright field (BF), green (PATZ1) and merge image of the insets. Scale bar: 100 μm. (**B**) Representative confocal fluorescence images on PFA-fixed cells stained with DAPI. Single fluorescence detection of either DAPI (blue) or PATZ1 (red) and digital merge of the images are shown. Colocalization (pink) of PATZ1 and DAPI is indicated by arrows. Negative control (CTRL) represented by untransfected cells is shown on the right. Scale bar: 10 μm.

We also transfected A549 and H1299 cells with the PATZ1-EGFP-C2 plasmid, carrying PATZ1 cDNA fused with enhanced green fluorescent protein [20], in order to observe its subcellular localization by fluorescence microscopy in living cells. As shown by merging images of bright field and green, PATZ1 appears to be expressed in both the nucleus and cytoplasm of both cell lines (Figure 6A). To better analyze the nuclear localization, we also stained PFA-fixed cells with DAPI and observed colocalization of PATZ1 and DAPI by confocal microscopy (Figure 6B).

### 3.5. PATZ1 Expression Attenuated EMT in NSCLC Cells

**Figure 7:**
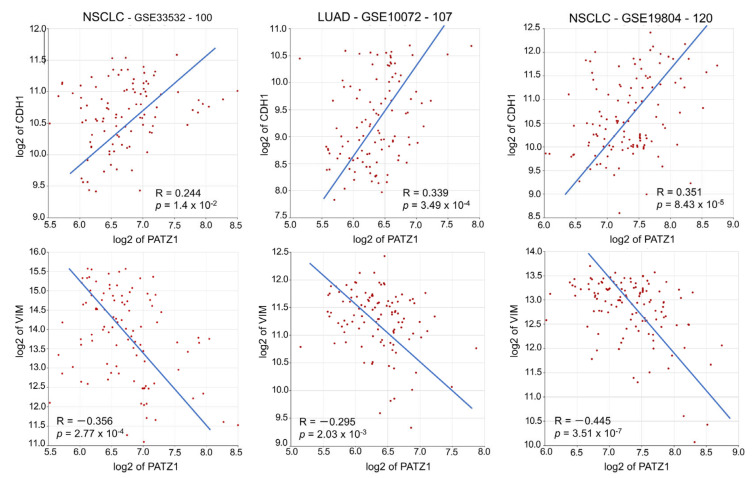
PATZ1 negatively correlates with EMT in NSCLC. XY-dot plots showing the correlation between PATZ1 (*X*-axis) and CDH1 (*Y*-axis) or VIM (*Y*-axis) gene expression in three publicly available gene expression NSCLC datasets analyzed via the R2: Genomic Analysis and Visualization platform [36].

It has been reported that in NSCLC cells, PD-L1 expression is induced by the epithelial to mesenchymal transition (EMT) [42]. We have previously shown that PATZ1 counteracts EMT in thyroid cancer cells [20]. To explore whether PATZ1 inhibits EMT and induces the reverse process, mesenchymal to epithelial transition (MET), also in NSCLC, we first analyzed the expression of PATZ1 and the EMT master genes CDH1 (E-cadherin) and VIM (Vimentin) in NSCLC tissues. For this purpose, we again used public datasets downloaded through the R2: Genomic Analysis and Visualization platform [36] and found that PATZ1 expression in these tumors significantly correlated positively with CDH1 and negatively with VIM (Figure 7).

**Figure 8:**
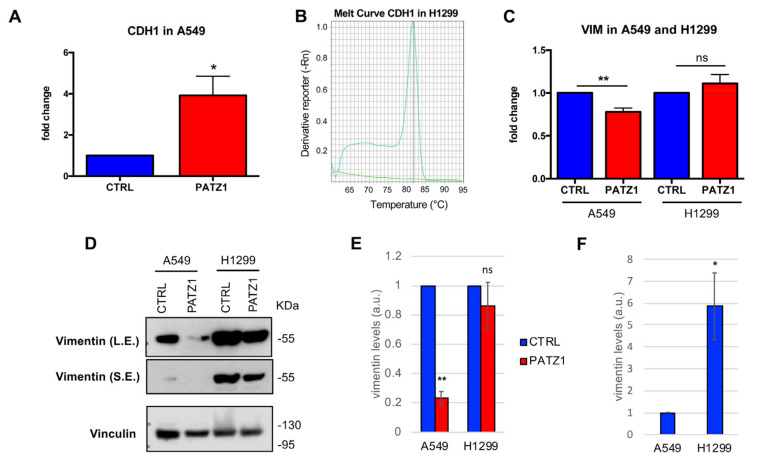
PATZ1 overexpression attenuates EMT in NSCLC cells. RT-qPCR (**A**–**C**) and Western blot (**D**–**F**) results in A549 and H1299 cells transfected with PATZ1-overexpressing plasmid (PATZ1) or empty vector used as control (CTRL). (**A**) Relative gene expression of CDH1 in A549 cells, as assessed by RT-qPCR. (**B**) Representative melting curves of CDH1 amplification in H1299 cells. A distinct curve (Tm = 81.66), indicative of a specific amplified transcript, was observed in PATZ1 (cyan) but not in CTRL (green) samples. Melting curves of the related actin amplification are shown in Figure S4. (**C**) Relative gene expression of VIM in A549 and H1299 cells. (**D**) Western blots using anti-vimentin on total cell extracts of A549 and H1299 cells. Vinculin was used for normalization. L.E., longer exposure; S.E., shorter exposure. (**E**) The relative levels of vimentin were quantified by Image J (v1.46r), normalized with respect to vinculin, and expressed relative to each control, arbitrarily set to 1. (**F**) The relative levels of vimentin in both control cells are shown. Mean values +/− SE of two or three independent experiments are reported in A, C, E, F. * *p* < 0.05; ** *p* < 0.01; ns, not significant. The uncropped blots are shown in Figure S5.

Then, we analyzed the expression of VIM and CDH1 in the two PATZ1-transfected NSCLC cell lines in comparison with empty vector-transfected controls (Figure 8 and Figure S4).

CDH1 was increased in both cell lines following overexpression of PATZ1 (panels A, B). In H1299 cells, a specific transcript was detected in PATZ1-overexpressing cells, while it was completely absent in control cells (panel B). On the other hand, VIM was downregulated in PATZ1 overexpressing A549 cells, while it was not significantly changed in H1299 cells (panel C). These latter results were confirmed at the protein level, showing that vimentin levels were markedly downregulated in PATZ1-transfected A549 cells compared to controls while they were unchanged in H1299 cells (panels D, E). Thus, overexpression of PATZ1 can attenuate EMT in NSCLC cells, with upregulation in the expression of the epithelial marker E-cadherin and concomitant downregulation of the mesenchymal marker vimentin. The absence of effects on vimentin in H1299 cells is probably due to the high levels of its basal expression in this cell line (panel F) in agreement with its mesenchymal phenotype [43].

### 3.6. PATZ1 Overexpression in A549 and H1299 NSCLC Cell Lines Inhibits Cell Proliferation, Migration, and Invasion

**Figure 9:**
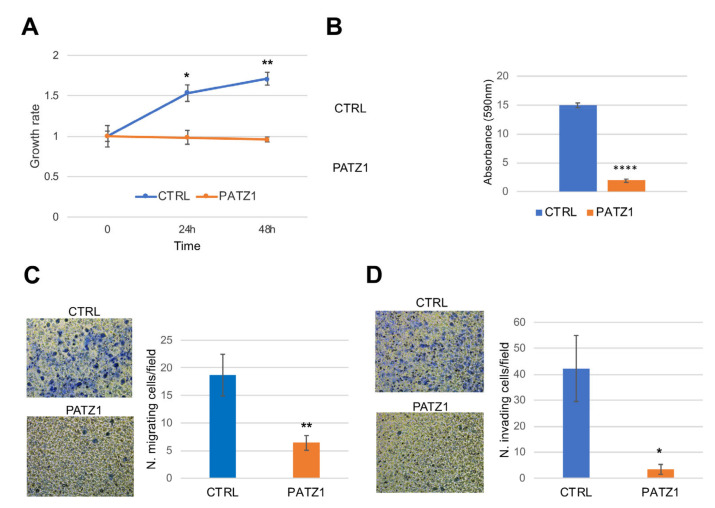
Functional analysis of PATZ1 overexpressing A549 NSCLC cells. The cells were transfected with a vector expressing PATZ1 cDNA or its corresponding empty vector. (**A**) Proliferation assay by cell viability analysis. Growth rate was calculated with respect to time 0 corresponding to 24 h post-transfection. (**B**) Colony-forming assays. Cells were plated 5 h post-transfection, cultured for 10 days in presence of G418, and stained with crystal violet. The color was eluted, and absorbance measured at 590 nm. A representative experiment is shown on the left of the graph. (**C**) Transwell migration assay. Representative images of a field of view (20× magnification) are shown on the left. Mean values +/− SE of 20 fields are shown. (**D**) Invasion transwell assays through a Matrigel layer. Representative images of a field of view (10× magnification) are shown on the left. Mean values +/− SE of 15 and 10 fields for CTRL and PATZ1, respectively, are shown on the right. All experiments were performed in triplicate. * *p* < 0.05; ** *p* < 0.01; **** *p* < 0.0001.

To investigate whether the overexpression of PATZ1 plays a role in NSCLC cancer cell function, we analyzed the proliferative, migratory, and invasive abilities of PATZ1- overexpressing A549 and H1299 cells compared to control cells (Figure 9 and Figure S6).

The growth rate was drastically reduced in PATZ1-overexpressing cells, as assessed by a cell viability assay (Panel A), suggesting an inhibitory role of PATZ1 overexpression on NSCLC cell proliferation. As a control, we stained transfected cells with trypan blue and did not detect significant numbers of dead cells in PATZ1-overexpressing cells compared with controls. However, we cannot exclude that sustained overexpression of PATZ1 beyond this time could induce cell death, but we experienced that stable high expression of PATZ1 is not achievable in these cells. To confirm the results of the cell viability assay, we also carried out colony-forming assays, which resulted for both cell lines in a remarkable decrease of the colony number in PATZ1 transfected cells compared to controls (Panel B). Next, using Boyden chambers, we analyzed migration across 8 μm membrane pores of starved cells in response to FBS (Panel C). Overexpression of PATZ1 caused a significant decrease in migrating cells (35% and 18% compared to controls in A549 and H1299 cells, respectively), indicating an inhibitory role of PATZ1 protein on NSCLC cell migration. As a control, we analyzed the cell viability of the starved cells, to exclude that the reduced number of migrating cells could be due to reduced proliferation (Figure S7). Finally, we repeated the same assay across a Matrigel-coated membrane, which mimics the entire process of invasion, including adhesion to a substrate, dissolution of the extracellular matrix, and migration [44]. As shown in Figure 8D for A549 cells and Figure S2D for H1299 cells, the invasive capacity of both cell lines was significantly impaired in PATZ1 overexpressing cells compared to empty vector- transfected controls. All together, these results indicate that PATZ1 has a key role in suppressing malignant features of NSCLC cells, suggesting a putative tumor suppressor role in this tumor type.

### 3.7. PATZ1 Knockout Can Induce LUAD in Mice

**Figure 10:**
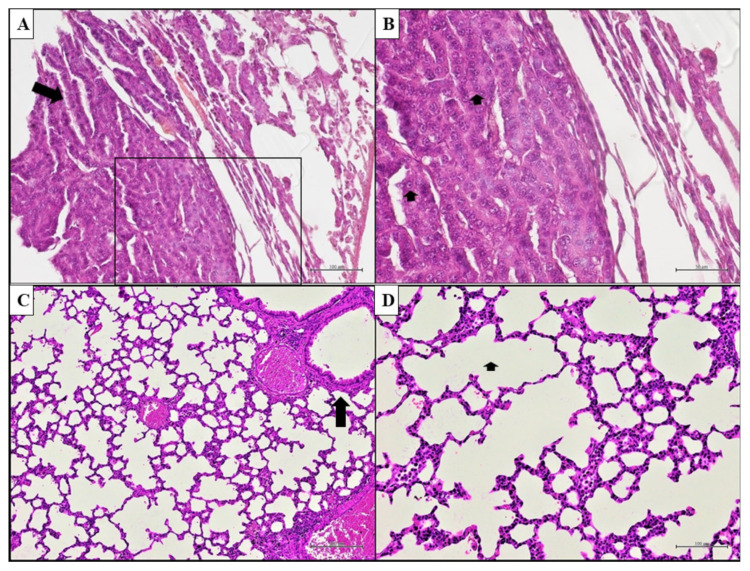
Lung adenocarcinomas in Patz1-knockout mice. (**A**) Representative LUAD with lepidic pattern (left arrow) growing along the surface of the alveolar air spaces of a Patz1^+/−^ mouse. The mass is composed of neoplastic cells with loss of polarity, irregularly placed overlapping nuclei, and marked nuclear atypia. (**B**) Higher-power image of the sample shown in A (inset area); better shows the marked pleomorphism and the nuclear atypia: neoplastic cells with nucleomegaly and increased nucleus-to-cytoplasm ratio; nuclei vesicular with fine chromatin and prominent nucleoli (arrowheads). (**C**) Representative normal lung parenchyma in a Patz1^+/+^ mouse. Alveolar spaces lined by type 1 and type 2 pneumocytes and a respiratory bronchiole with adjacent arterioles (arrow) are shown. (**D**) Higher magnification of the alveolar epithelium from another area of the same Patz1^+/+^ sample shows lung alveoli lined by type 1 and type 2 pneumocytes with poor lymphocytic infiltrate in the alveolar septa (arrow). Hematoxylin & eosin. Scale bars: 50 μm in (**B**); 100 μm in (**A**,**D**); 200 μm in (**C**).

We previously generated knockout mice for the PATZ1 gene [35]. Both homozygous and heterozygous Patz1-knockout mice developed a range of malignancies, including lymphomas, hepatocellular carcinomas, sarcomas, and, rarely, lung adenocarcinomas (1 of 36 Patz1^+/−^ and 1 of 10 Patz1^−/−^) [9]. Here, we thoroughly analyzed the tumor pulmonary phenotype. It was characterized by neoplastic cells with loss of polarity, irregularly placed overlapping nuclei, and marked nuclear atypia, compatible with the diagnosis of lepidic LUAD (Figure 10). The development of LUADs in Patz1-knockout mice is consistent with the envisaged tumor suppressor role of PATZ1 in NSCLC.

**Figure 11:**
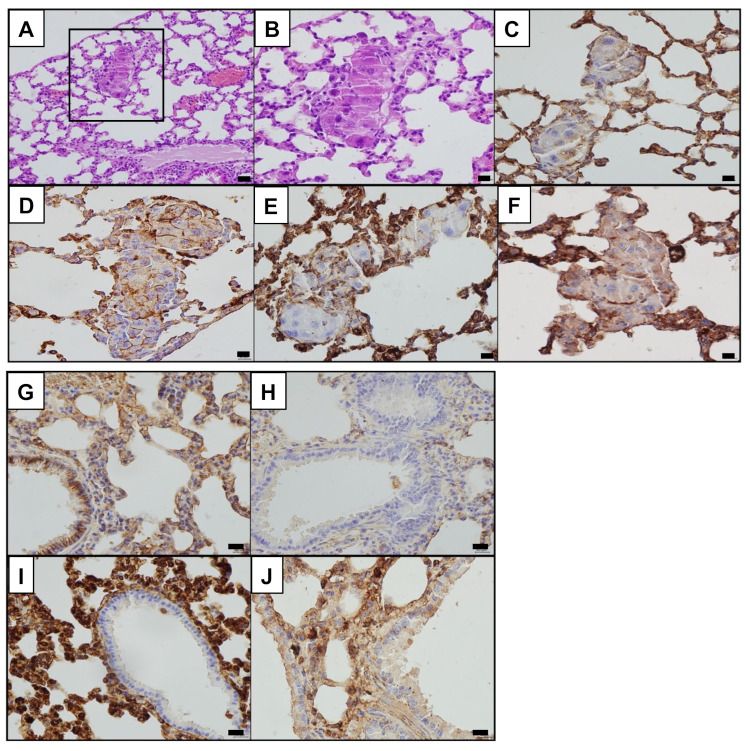
Immunohistochemical staining for EMT markers and PD-L1 in Patz1-knockout and wild-type control mice. (**A**–**F**) Representative specimens from a Patz1^+/−^ mouse; (**G**–**J**) representative specimens from a Patz1^+/+^ mouse. (**A**) Hematoxylin & eosin showing a nest of subpleural LUAD in mildly emphysematous lung. (**B**) Higher magnification of the inset area shown in (**A**). LUAD is characterized by pleomorphic cells with atypical nuclei, nucleomegaly, and focal and evident nucleoli. (**C**) E-cadherin immunohistochemical staining showed lack of expression in neoplastic cells. (**D**) Widespread membrane expression of N-cadherin in LUAD cells. (**E**) Patchy membrane and cytoplasmic expression of vimentin in LUAD cells. (**F**) Membrane expression of PD-L1 in LUAD and inflammatory cells of the inter-alveolar septa. (**G**) Evidence of E-cadherin membrane expression in bronchiolar epithelium and in some alveolar cells. (**H**) Complete lack of N-cadherin expression in bronchiolar epithelium and lung parenchyma. (**I**) Diffuse cytoplasmic vimentin immunostaining in stromal cells, as fibroblasts and inflammatory cells of the inter-alveolar septa, in contrast to complete lack of vimentin expression in bronchiolar cells. (**J**) Lack of PD-L1 expression in bronchiolar epithelium and alveolar cells with focal membrane protein expression in inflammatory cells of the inter-alveolar stroma. Scale bars: 100 μm in (**A**); 50 μm in (**B**–**J**).

On the same mouse samples, we also conducted immunohistochemical analysis to evaluate in vivo the negative role of PATZ1 on EMT and PD-L1 in lung adenocarcinoma cells. As shown in Figure 11, we confirmed induction of EMT and PD-L1 expression in Patz1^+/−^ LUAD samples, as demonstrated by the absence of E-cadherin expression and positive staining for N-cadherin, vimentin, and PD-L1 (Figure 11C–F). In contrast, the lung epithelium from Patz1^+/+^ control mice was positive for E-cadherin and negative for N-cadherin, vimentin, and PD-L1 (Figure 11G–J).

## 4. Discussion

Traditionally and still according to the current WHO classification of lung cancer, non-small cell lung cancer (NSCLC) includes any type of lung cancer other than small cell lung cancer (SCLC), which can be identified through its neuroendocrine features [45]. However, experimental and clinical evidence is accumulating that indicates significant heterogeneity among NSCLC subtypes, as recently confirmed by transcriptomic analyses associated with prognostic markers, genetic drivers, histopathology, biological history, and clinical outcome [46]. Indeed, whole-transcriptome profiling indicated profound diversity in LUAD versus LUSC, suggesting they should be classified as distinct diseases [46,47]. Intriguingly, systematic analysis of mutated tumor genes identified EGFR, MET, BRAF, and TERT as determinants in LUAD, and NOTCH, FGFR1, SOX2, and PI3CA as determinants in LUSC [48]. Among these genes, the known stem cell marker SOX2 is the determining oncogenic switch in promoting LUSC from different cells of origin [49], suggesting distinct tumor progression trajectories for these two NSCLC subtypes [46]. Indeed, LUSC seems to originate from both stem cells and progenitors (basal, secretory, or cuboidal type 2 (AT2) cells), where SOX2 overexpression promotes squamous differentiation in an otherwise non-squamous epithelium, in conjunction with other oncogenic hits [50]. In this context, PATZ1 overexpression in LUSCs appears consonant with the stem cell phenotype of this subtype. In fact, PATZ1 is required for pluripotency maintenance of embryonic stem cells [51], plays an essential role in the reprogramming of differentiated cells toward stem cells [13], and governs the self-renewal ability of adult stem cells, including neural and cancer stem cells [52,53]. Strikingly, the expression of PATZ1 and the stem cell marker SOX2 are positively associated in NSCLC tissues (Figure S8). Therefore, it is likely that high PATZ1 expression contributes to the stem cell signature, which characterizes the LUSC subtype. It is noteworthy that, as we previously observed for proneural GBM subtype associated with a stem cell signature [28], within the LUSC subtype, higher levels of PATZ1 predict a better survival. It is likely that in these tumors, PATZ1 upregulation may be a mechanism of defense to protect cellular homeostasis against acquiring malignant features and full transformation. This role could be also extended to LUAD, where PATZ1 is not upregulated in tumors compared to normal tissue, but lower levels of PATZ1 are predictive of a worse survival. In support of this hypothesis, we show evidence in vitro and in vivo that PATZ1 acts as a tumor suppressor of NSCLC. Indeed, Patz1-knockout mice develop NSCLC, and overexpression of PATZ1 in NSCLC cells attenuates EMT and inhibits their proliferation, migration, and invasion, as previously observed in thyroid cancer cells [20]. Future studies are needed to focus on the molecular mechanisms that involve PATZ1 in each of these important cellular functions that contribute to lung cancer progression. It is well known in other cell contexts that PATZ1 has a role in both cell cycle arrest and senescence. In fact, Patz1^−/−^ mouse embryonic fibroblasts (MEFs) grow slower than wild-type controls, showing cell cycle defects, premature senescence, and increased protein expression of cell cycle regulators, including both inhibitors (i.e., p53, p21, p27, p16) and activators (i.e., CDK4, cyclin D2, HMGA1/2). These conflicting signals could be responsible for a hyper mitogenic arrest, which in turn induces premature senescence [37]. Downregulation of PATZ1 in human young endothelial cells also induces premature senescence [12], and PATZ1 is among the differentially expressed genes that characterize the senescence core signature [54]. On the other hand, Patz1^+/−^ MEFs express lower levels of the gene encoding the cell cycle inhibitor p21 [10] and are not senescent [13].

Only a few studies have so far analyzed how PATZ1 is regulated [55,56]. One of them revealed a post-transcriptional regulation by the microRNA miR-24, which downregulates PATZ1 expression [56]. Interestingly, miR-24 has been reported to be a good biomarker of NSCLC [57], playing a key role in its biology. In particular, the downregulation of miR-24 significantly reduces the proliferation, migration, and invasion of A549 and H1299 cells [58]. Based on the results shown here, we could speculate that PATZ1 downregulation may contribute to the protumorigenic role of miR-24 in NSCLC. Further studies will evaluate this possibility. Other studies focused on the regulation of PATZ1 nuclear/cytoplasm delocalization. In human testes and testicular seminomas [59], PATZ1 interacts with ERβ in the nucleus when ERβ is highly expressed, while it is delocalized in the cytoplasm when ERβ is down-regulated. The sequestration of PATZ1 in the cytoplasm is regulated by the cAMP pathway that, when activated, allows PATZ1 to translocate to the nucleus and transactivate its target genes. These results are consistent with previous work in normal fibroblasts, showing that PATZ1 interacts in the cytoplasm with the regulatory subunit (RIα) of cAMP-dependent protein kinase A (PKA) and suggesting that RIα may sequester PATZ1 in the cytoplasm, thereby regulating its transcriptional activity and function in cell growth control in response to cAMP [60]. Interestingly, it has been very recently proved that activation of the cAMP pathway impairs cell growth and migration ability of A549 and H1299 cells, where changes in cell-cycle progression and epithelial–mesenchymal markers were observed [61]. Future studies should investigate whether PATZ1 shuttling from cytoplasm to nucleus occurs upon cAMP activation in these cells.

In lung cancer patients, tumor PD-L1 expression levels have attracted particular interest because NSCLC patients show higher expression levels and tend to respond more favorably to evolving PD-1 and PD-L1 inhibitors [5]. By dividing LUSCs into tiers of PD-L1 expression level, Pawelczyk and colleagues found 64% of cases with low expression levels (<1%), 24.9% with moderate expression levels (1–49%), and 11.3% with high expression levels (≥50%) [62], inversely fitting with our PATZ1 expression levels in LUSCs (Table 4). Indeed, a pooled analysis of 9 published studies determined that PD-L1 tumor cell staining ≥1% is associated with significantly higher clinical activity of anti-PD-1/PD-L1 monoclonal antibodies in pre-treated NSCLC patients, suggesting a potential role of PD-L1 expression as a predictive biomarker for the selection of patients to be treated with immune-checkpoint inhibitors [63]. On the other hand, tumor expression of PD-L1 can promote NSCLC progression by both intrinsic and extrinsic mechanisms. Intrinsically, PD-L1 promotes in vitro cell proliferation, migration, and invasion, and in vivo tumor growth of NSCLC cells [8]. Extrinsically, it blocks T-cell-mediated immune response against the tumor, favoring immune evasion and consequently tumor growth [7]. In both cases, a negative regulator of PD-L1 could be a potential tumor suppressor, as is the case of PATZ1. Future studies will be aimed at investigating if the different PATZ1 expression in LUSC and LUAD tissues correlates with different immune responses and immune-cell infiltration. Recent studies demonstrated that human primary NSCLC tumors have reduced Th1 lymphocytes, are Th2-skewed, and contain numerous Treg cells [64]. Strikingly, T cell-specific depletion of PATZ1 leads to increased frequency of Treg cells, while enforced PATZ1 expression impairs Treg cell differentiation [65], suggesting that PATZ1 might regulate the carcinogenic process at both the cancer cell and microenvironment level.

Further research in our laboratory will examine the possibility that PATZ1 may bind to the CD274 promoter and regulate its activity to repress PD-L1 expression in NSCLC cells. Preliminary exploration of the PATZ1 consensus sequences on the CD274 promoter region by the TraFaC (Transcription Factor binding site Comparison) webtool [66] yielded no positive results. However, PATZ1 could also bind to AT-rich sequences by means of its AT-hook or be recruited to the promoter through interaction with other proteins. Another possibility to be taken in consideration is that PATZ1 could regulate PD-L1 by inhibiting EMT. In fact, PD-L1 is induced by EMT in NSCLC cells [42], and here we show that PATZ1 expression, which is inversely correlated to EMT markers in NSCLC tissues, can inhibit EMT in NSCLC cells. Therefore, a PATZ1/EMT/PD-L1 axis could be involved in NSCLC.

However, independently from the mechanisms by which PATZ1 is involved in the negative regulation of PD-L1 expression, its role could be exploited for therapeutic purposes. Indeed, targeting a negative regulator of PD-L1 is a promising therapeutic approach for NSCLC with acquired resistance to immunotherapy targeting the PD-1/PD-L1 axis. Therefore, if the causal role of PATZ1 in the downregulation of tumor PD-L1 expression is confirmed, future work should investigate the possibility that targeting PATZ1 could enhance the efficacy of anti-PD-1/PD-L1 immunotherapy for NSCLC. According to this idea, in a Kaplan–Meier PFS analysis of a cohort of immune-treated PAN-cancer patients, high PATZ1 levels correlated with a worse outcome with a median survival of 5 months versus 15.7 months in the low-expression cohort (Figure S9). Therefore, it is possible to extend the role of PATZ1 on PD-L1 expression also in cancers other than NSCLC.

## 5. Conclusions

In conclusion, we observed that PATZ1 expression and subcellular localization are regulated in NSCLC, where high expression and nuclear localization are associated with the LUSC subtype, low tumor PD-L1 expression, and, at least for gene expression, better survival, suggesting that it could be used as a diagnostic and prognostic biomarker of this disease. Furthermore, we demonstrated that overexpression of PATZ1 in NSCLC cells attenuates EMT and inhibits malignant features, such as cell proliferation, migration, and invasion, thus suggesting a tumor suppressor role for PATZ1. This was supported by our in vivo studies showing the development of NSCLC in PATZ1 gene knockout mice, in which EMT and PD-L1 expression were induced.

## Figures and Tables

**Table 1:** Clinicopathological characteristics of the tissue micro-array.

Biomarker		Tot (n = 104)	Subtype and Grade		Stadium	
Gender	F	29 (27.9%)	LUSC	8		
			LUAD	21		
	M	75 (72.1%)	LUSC	35		
			LUAD	40		
Median Age	65.6 ± 2.77		LUSC	66.5 ± 3.20		
			LUAD	65.2 ± 2.64		
Subtype, grade and stadium	LUSC	43 (41.3%)	G1	2	IA1	4
			G2	27	IA2	11
			G3	14	IB	7
					IIA	8
					IIB	8
					IIIA	4
					IIIB	1
	LUAD	61 (58.7%)	G1	19	IA1	3
			G2	29	IA2	12
			G3	13	IB	23
					IIA	10
					IIB	5
					IIIA	6
					IIIB	2

**Table 2:** PATZ1 expression and sub-cellular localization in NSCLC and adjacent non-tumor epithelium.

Subtype ^1^	N.	PATZ1 Sub-Cellular Localization ^2^			Negative
		N	N/C	C	
LUSC	43	15 (35%)	16 (37%)	7 (16%)	5 (12%)
LUAD	61	7 (11%)	29 (47%)	18 (29%)	7 (11%)
Bronchial epithelium	4	1 (25%)	3 (75%)	-	-
Alveolar epithelium	30	16 (53%)	-	-	14 (47%)

^1^ including LUSC, lung squamous cell carcinoma; LUAD, lung adenocarcinoma, and adjacent non-tumor epithelium. ^2^ N, nuclear; N/C, nuclear and cytoplasmic; C, cytoplasmic.

**Table 3:** Contingency table showing the correlation between PATZ1 nuclear expression and LUSC/LUAD subtypes ^1^.

		Nuclear PATZ1		*p*	R
		(−)	(+)		
LUSC	(−)	47 (87%)	7 (13%)	0.003	0.306
	(+)	23 (60.5%)	15 (39.5%)		
LUAD	(−)	23 (60.5%)	15 (39.5%)	0.003	−0.306
	(+)	47 (87%)	7 (13%)		

^1^ The table shows total number (and percentage values in brackets) of samples double positive or single positive for each LUSC or LUAD subtype (rows) and nuclear PATZ1 expression (columns). Data were analyzed by Pearson’s χ^2^ test, and the resulting *p* value and R coefficient are reported where indicated.

**Table 4:** PATZ1 expression groups based on the percentage of stained cells.

Subtype ^1^	Low PATZ1 (Absent or <20%)				Medium PATZ1 (≥20%–<70%)			High PATZ1 (≥70%)		
	(−)	N	N/C	C	N	N/C	C	N	N/C	C
LUSC	5 (11%)	2 (5%)	2 (5%)	2 (5%)	11 (26%)	9 (21%)	5 (11%)	2 (5%)	5 (11%)	0
LUAD	7 (11%)	1 (2%)	0	5 (8%)	5 (8%)	28 (46%)	13 (21%)	1 (2%)	1 (2%)	0

^1^ LUSC, lung squamous cell carcinoma; LUAD, lung adenocarcinoma; (−), no expression; N, nuclear expression; N/C, nuclear and cytoplasmic expression; C, cytoplasmic expression.

**Table 5:** Contingency table showing the correlation between High PATZ1 and NSCLC subtype ^1^.

		PATZ1 ≥ 70%		*p*	R
		(−)	(+)		
LUSC	(−)	58 (97%)	2 (3%)	0.022	0.226
	(+)	36 (84%)	7 (16%)		
LUAD	(−)	36 (84%)	7 (16%)	0.022	−0.226
	(+)	58 (97%)	2 (3%)		

^1^ The table shows total number (and percentage values in brackets) of samples double positive or single positive for each LUSC or LUAD subtype (rows) and PATZ1 expression in ≥70% neoplastic cells (columns). Data were analyzed by Pearson’s χ^2^ test, and the resulting *p* value and R coefficient are reported where indicated.

**Table 6:** Contingency table showing the correlation between High PATZ1 and PD-L1 ^1^.

		PD-L1		*p*	R
		(−)	(+)		
High PATZ1	(−)	40 (47%)	45 (53%)	0.029	−0.227
	(+)	7 (87.5%)	1 (12.5%)		

^1^ The table shows total number (and percentage values in brackets) of samples double positive or single positive for PD-L1 expression (columns) and PATZ1 expression in ≥70% neoplastic cells (rows). Data were analyzed by Pearson’s χ^2^ test, and the resulting *p* value and R coefficient are reported where indicated.

## Data Availability

Publicly available datasets were analyzed in this study. These data can be found here: www.kmplot.com; http://r2.amc.nl; www.proteinatlas.org [33,36,41].

## Supplementary Materials

The following supporting information can be downloaded at: https://www.mdpi.com/article/10.3390/cancers15072190/s1, Figure S1: survival analysis in patients affected by lung cancer; Figure S2: correlation analysis between PATZ1 and PDCD1; Figure S3: original uncropped images of Western blots for Figure 5A; Figure S4: amplification plot and melting curves of actin of the qPCR shown in Figure 8A,B; Figure S5: original images of Western blots for Figure 8D; Figure S6: functional analysis of PATZ1-overexpressing H1299 NSCLC cells; Figure S7: PATZ1 overexpression does not inhibit cell proliferation in starved NSCLC cells; Figure S8: PATZ1 and SOX2 expression positively correlate in NSCLC; Figure S9: high expression of PATZ1 gene is associated with a worse progression-free survival (PFS) after immunotherapy.
