# Supplementary material for: PATZ1 in Non-Small Cell Lung Cancer: A New Biomarker That Negatively Correlates with PD-L1 Expression and Suppresses the Malignant Phenotype
Source: Cancers (Basel). 2023 Apr 6;15(7):2190. doi: 10.3390/cancers15072190 (PMC10093756; doi:10.3390/cancers15072190)
Supplement: Supplementary file 1 [file cancers-15-02190-s001.zip › cancers-2243928-supplementary.pdf]

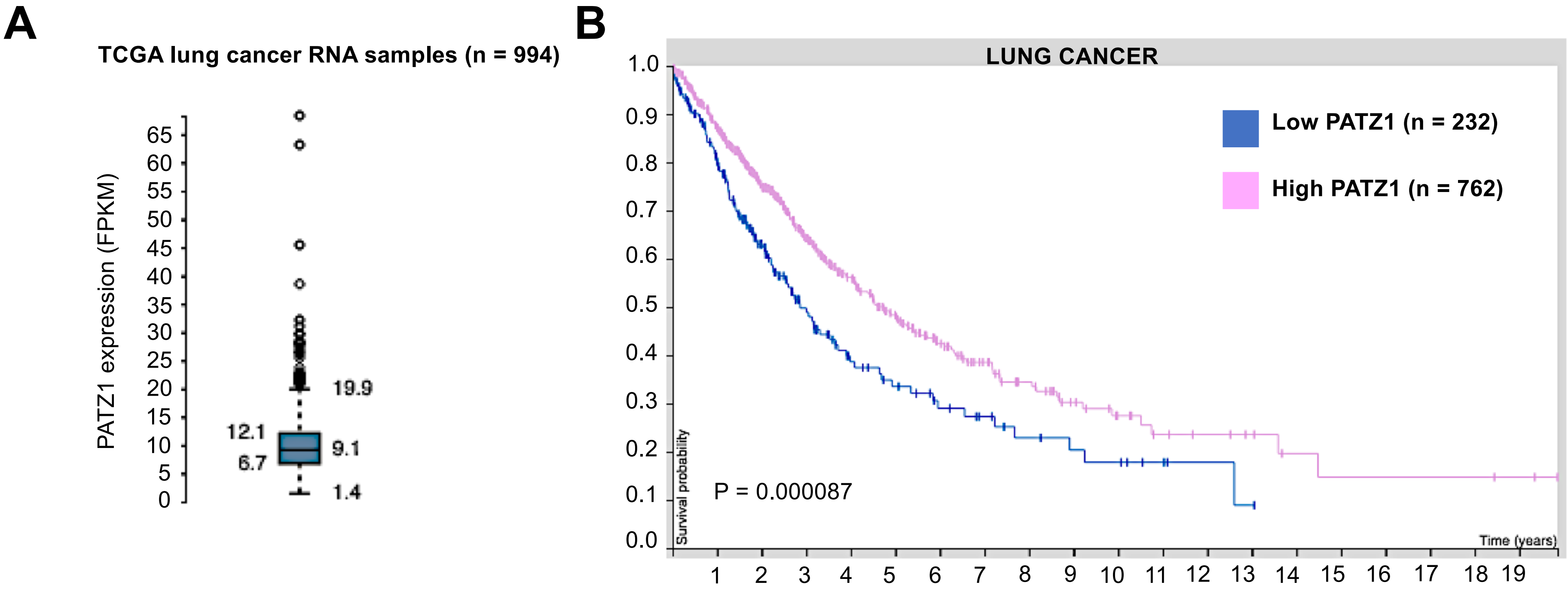

Figure S1. Survival analysis in patients affected by lung cancer. (**A**) TCGA lung cancer RNA samples were analyzed by RNAseq for PATZ1 expression through the the Human Protein Atlas web platform, subsection "pathology" [41]. Median expression was 9.1 FPKM (fragments per kilobase of exon per million mapped fragments). (**B**) The graph shows survival probability based on PATZ1 expression (cut off: 6.55 FPKM). The results indicate that PATZ1 is prognostic, high expression is favorable in lung cancer.

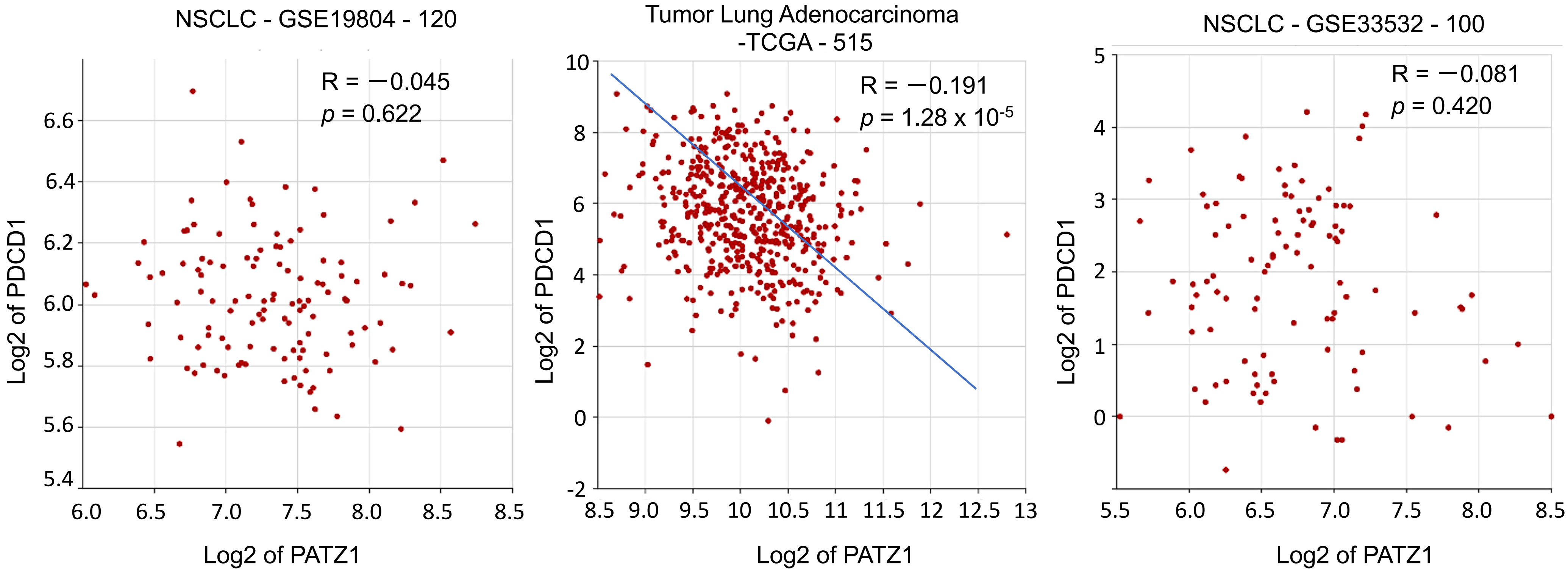

Figure S2. Correlation analysis between PATZ1 and PDCD1. XY-dotplots showing the correlation between PATZ1 (X-axis) and PDCD1 (Y-axis) gene expression in three publicly available NSCLC gene expression datasets analyzed via the R2: genomic Analysis and Visualization platform [36]. The correlation was significant only in the LUAD TCGA dataset (middle panel).

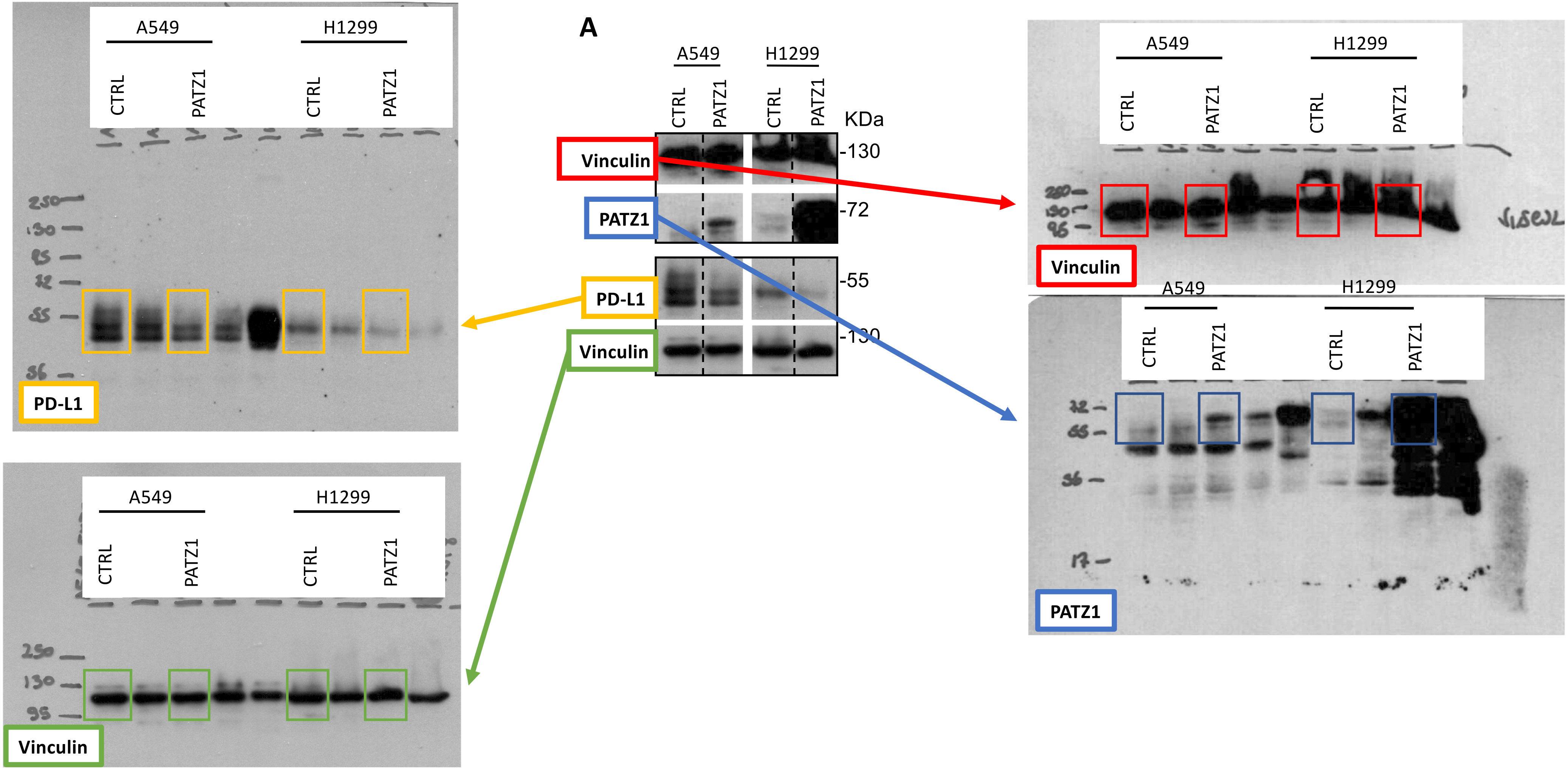

Figure S3. Original uncropped images of western blots for Figure 5A

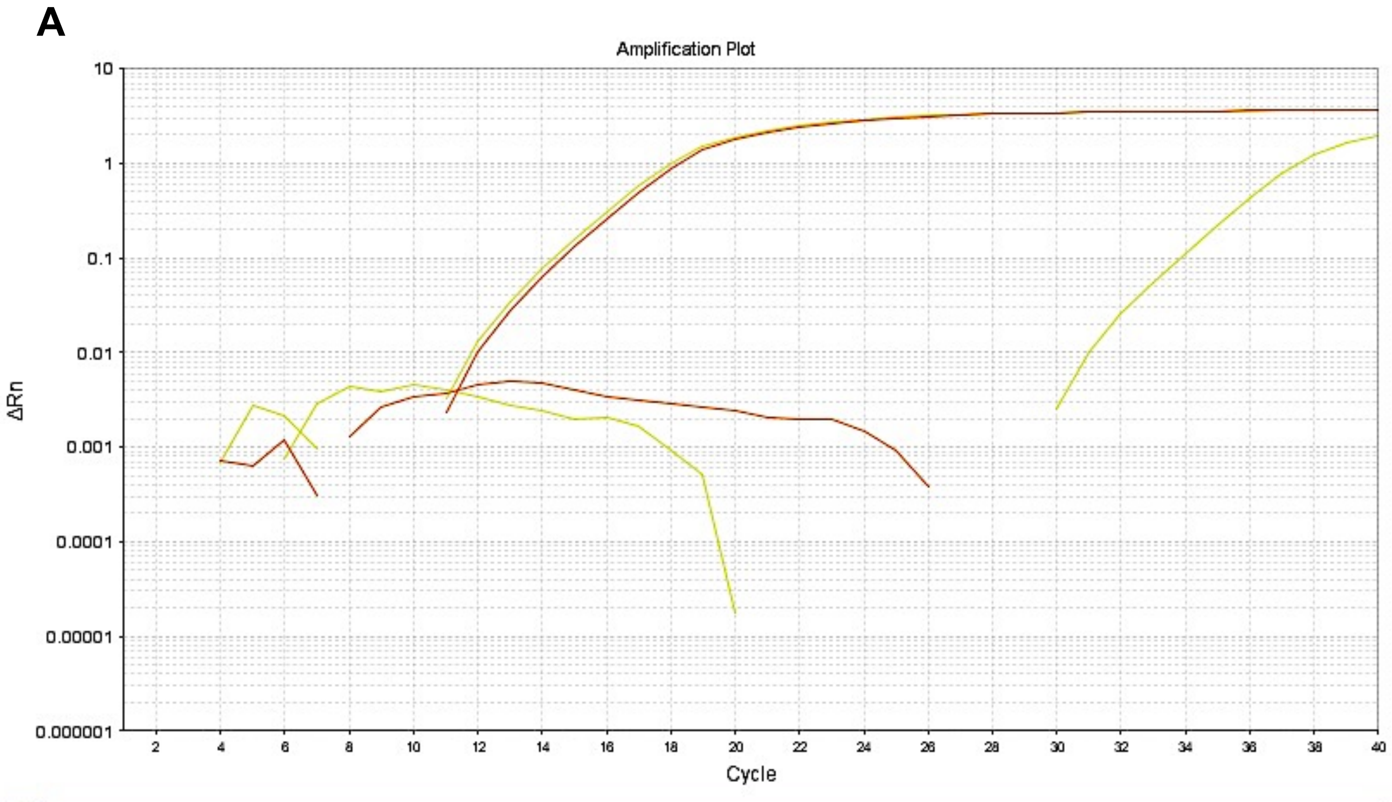

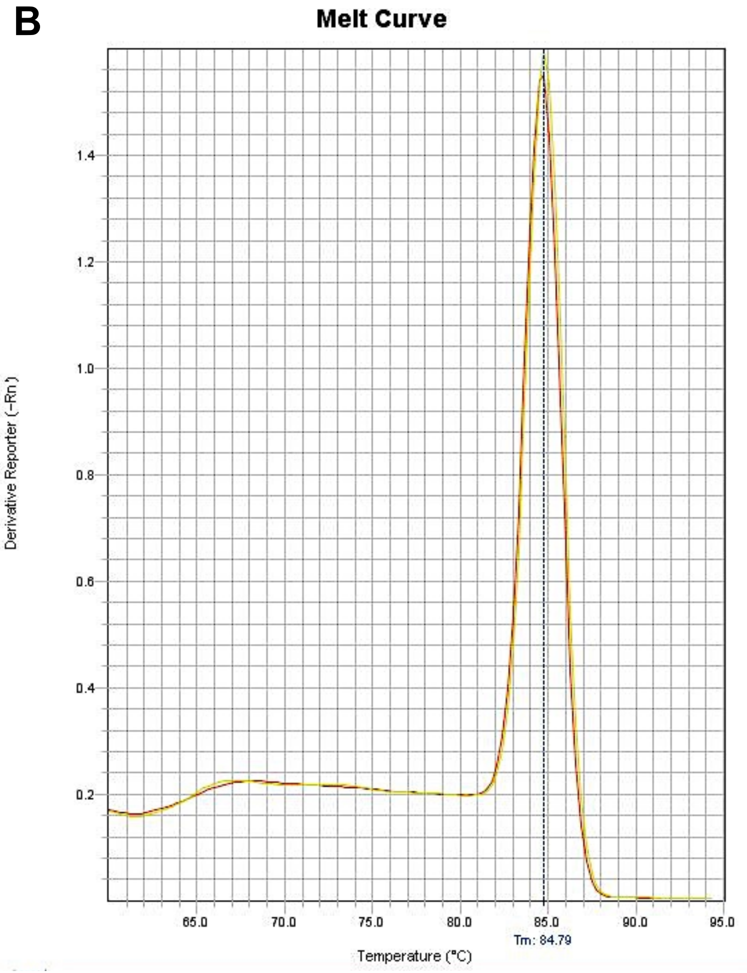

Figure S4. (**A**) Representative amplification plot of CDH1 and beta-actin (internal control) in PATZ1-overexpressing (yellow) and control (red) H1299 cells. (**B**) Melting curves for beta-actin amplification. A distinct curve (Tm = 84.79), indicative of a specific amplified transcript, was observed in both samples.

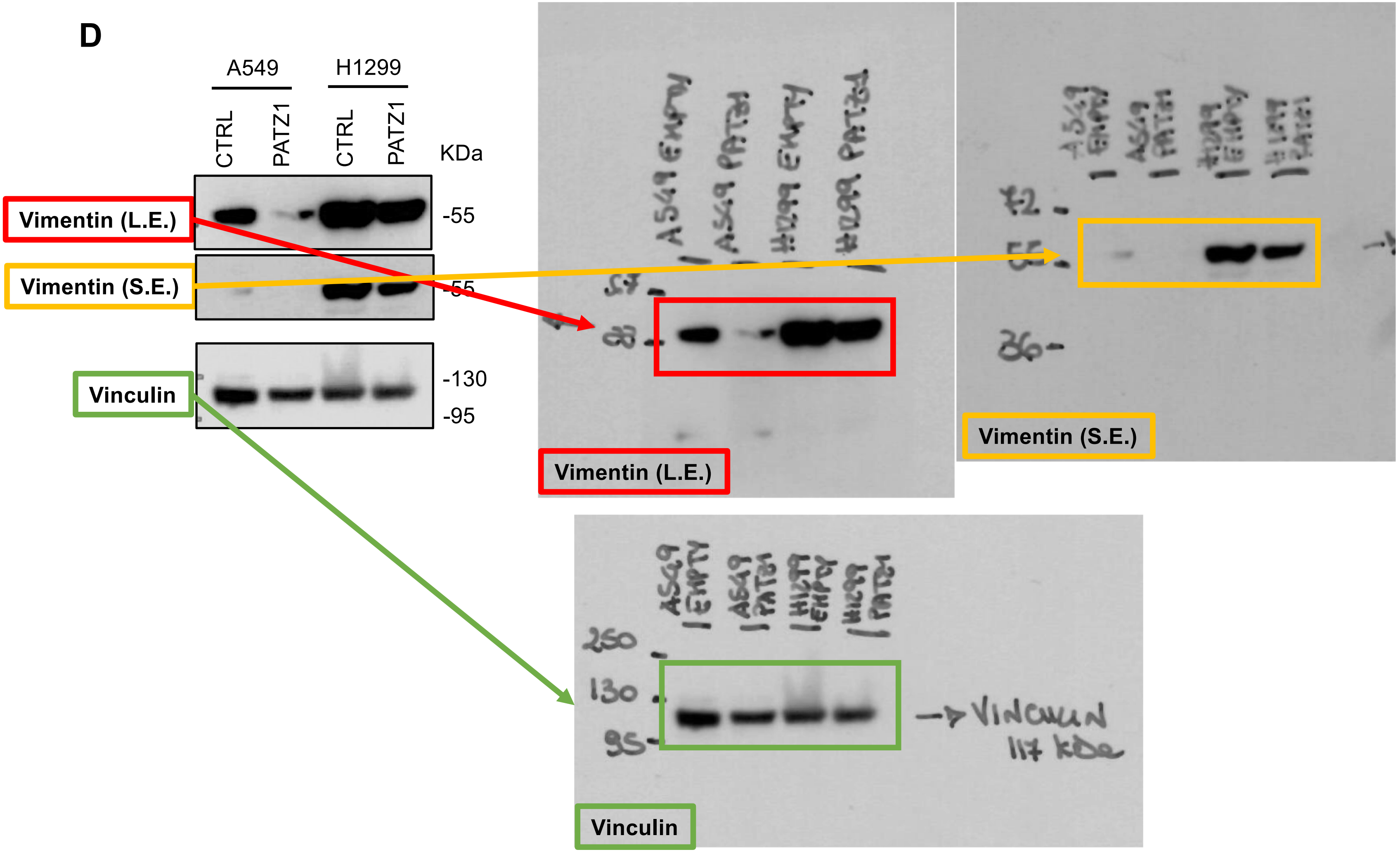

Figure S5. Original images of western blots for Figure 8D

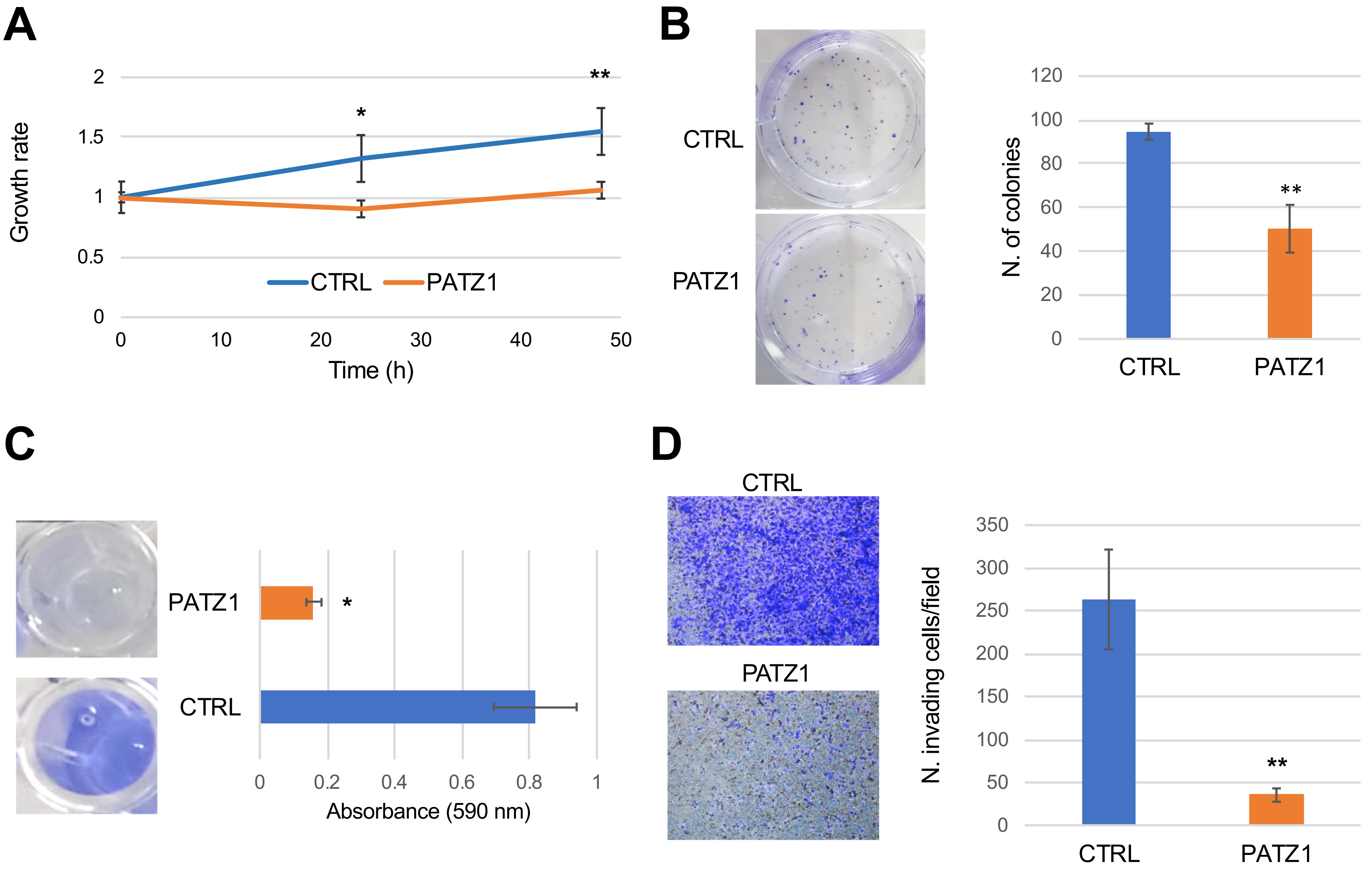

Figure S6. Functional analysis of PATZ1 overexpressing H1299 NSCLC cells. The cells were transfected with a vector expressing PATZ1 cDNA or its corresponding empty vector. (**A**) Proliferation assay by cell viability analysis. Growth rate was calculated with respect to time 0 corresponding to 24 h post-transfection. (**B**) Colony-forming assays. Cells were plated 5h post-transfection, cultured for 10 days in presence of G418, and stained with crystal violet. The average number of colonies +/— SE was reported in the bar graph. A representative experiment is shown on the left of the graph. (**C**) Transwell migration assay. A representative experiment is shown on the left. The color was eluted, and absorbance measured at 590nm. Mean values +/— SE are shown on the right. (**D**) Invasion transwell assays through a Matrigel layer. Representative images of a field of view (10× magnification) are shown on the left. Mean values +/— SE of 15 and 10 fields are shown on the right for CTRL and PATZ1, respectively. All experiments were performed in triplicate. *, $p < 0.05$; **, $p < 0.01$.

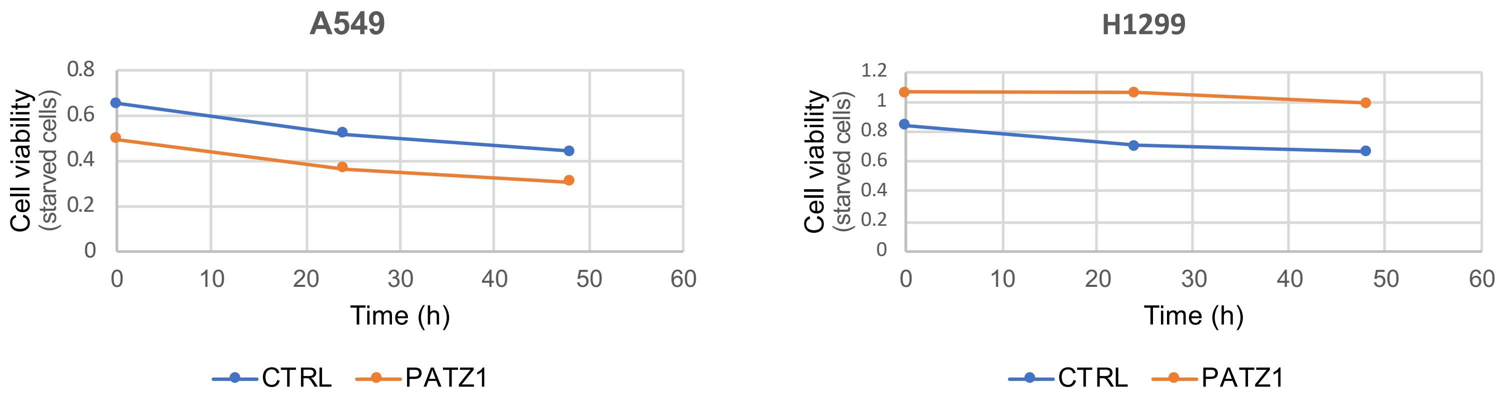

Figure S7. PATZ1 overexpression does not inhibit cell proliferation in starved NSCLC cells. Cell viability assay was carried out in parallel with the transwell assay. No differences were detected between PATZ1-transfectants and controls in both A549 and H1299 cell lines.

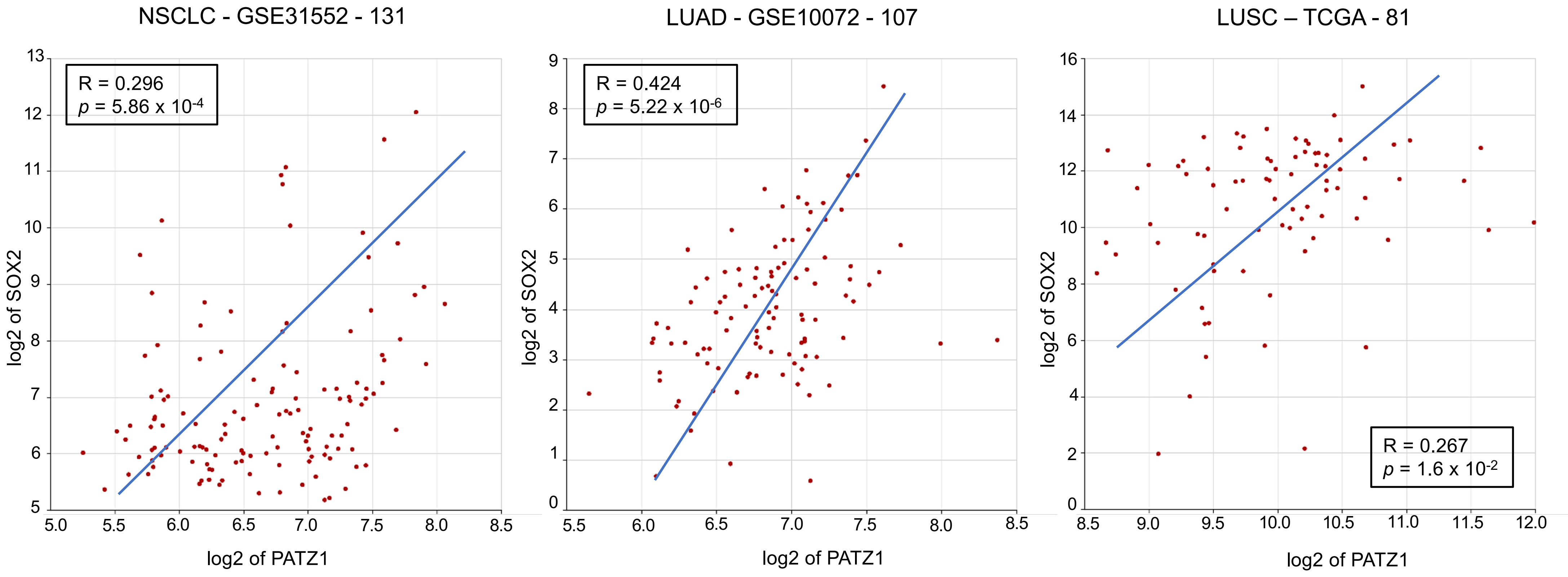

Figure S8. PATZ1 and SOX2 expression positively correlate in NSCLC. XY-dotplots showing the correlation between PATZ1 (X-axis) and SOX2 (Y-axis) gene expression in three publicly available gene expression NSCLC datasets analyzed via the R2: genomic Analysis and Visualization platform [36].

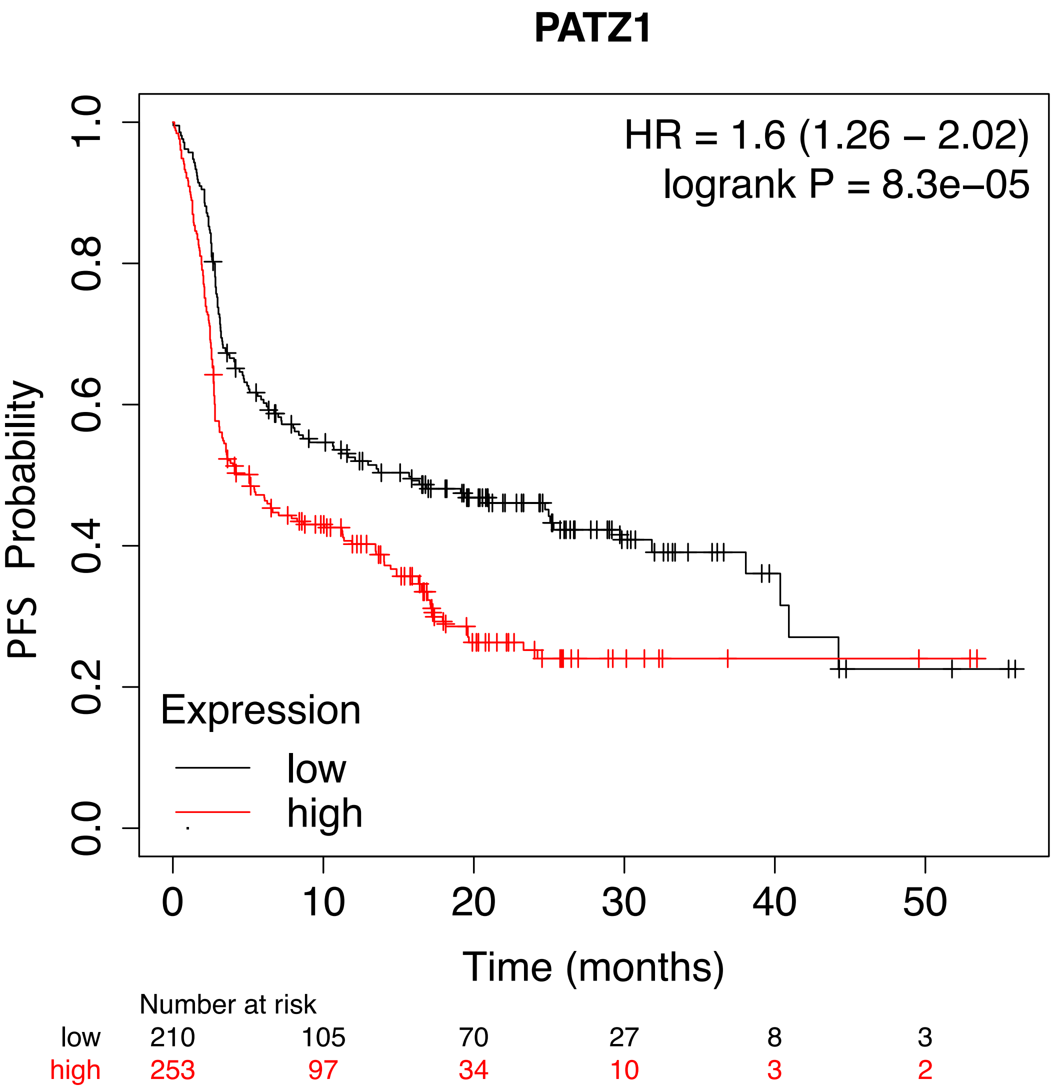

Figure S9. High expression of PATZ1 gene is associated with a worse progression-free survival (PFS) after immunotherapy. Kaplan-Meier curves in a cohort of PAN-cancer patients using publicly available transcriptomic and clinical data via the Kaplan-Meier Plotter platform [33]. The expression cutoff was set to 224 (close to the median) according to the scan function. HR, Hazard ratio.
